# Expression of guanylate cyclase C in human prefrontal cortex depends on sex and feeding status

**DOI:** 10.3389/fnmol.2024.1361089

**Published:** 2024-05-22

**Authors:** Martina Ratko, Vladiana Crljen, Martina Tkalčić, Anton Mažuranić, Pero Bubalo, Petar Škavić, Ivan Banovac, Aleksandra Dugandžić

**Affiliations:** ^1^Laboratory for Cellular Neurophysiology, Croatian Institute for Brain Research, School of Medicine, University of Zagreb, Zagreb, Croatia; ^2^Centre of Excellence for Basic, Clinical and Translational Neuroscience, School of Medicine, University of Zagreb, Zagreb, Croatia; ^3^Department of Physiology, School of Medicine, University of Zagreb, Zagreb, Croatia; ^4^Institute for Forensic Medicine, School of Medicine, University of Zagreb, Zagreb, Croatia; ^5^Department of Anatomy and Clinical Anatomy, School of Medicine, University of Zagreb, Zagreb, Croatia

**Keywords:** human prefrontal cortex, cerebellum, hypothalamus, substantia nigra, feeding

## Abstract

**Introduction:**

Guanylate cyclase C (GC-C) has been detected in the rodent brain in neurons of the cerebral cortex, amygdala, midbrain, hypothalamus, and cerebellum.

**Methods:**

In this study we determined GC-C protein expression in Brodmann areas (BA) 9, BA10, BA11, and BA32 of the human prefrontal cortex involved in regulation of feeding behavior, as well as in the cerebellar cortex, arcuate nucleus of hypothalamus and substantia nigra in brain samples of human 21 male and 13 female brains by ELISA with postmortem delay < 24 h.

**Results:**

GC-C was found in all tested brain areas and it was expressed in neurons of the third cortical layer of BA9. The regulation of GC-C expression by feeding was found in male BA11 and BA10-M, where GC-C expression was in negative correlation to the volume of stomach content during autopsy. In female BA11 there was no correlation detected, while in BA10-M there was even positive correlation. This suggests sex differences in GC-C expression regulation in BA11 and BA10-M. The amount of GC-C was higher in female BA9 only when the death occurred shortly after a meal, while expression of GC-C was higher in BA10-O only when the stomach was empty. The expression of GC-C in female hypothalamus was lower when compared to male hypothalamus only when the stomach was full, suggesting possibly lower satiety effects of GC-C agonists in women.

**Discussion:**

These results point toward the possible role of GC-C in regulation of feeding behavior. Since, this is first study of GC-C regulation and its possible function in prefrontal cortex, to determine exact role of GC-C in different region of prefrontal cortex, especially in humans, need further studies.

## Introduction

According to the World Health Organization (WHO), each year 4 million people die due to obesity. The prefrontal cortex (PFC) is involved in the regulation of human feeding behavior (Lowe et al., 2019). The areas of the PFC that play a role in feeding regulation are the dorsolateral, ventromedial and orbitofrontal cortex (Lowe et al., 2019; Rolls, 2023). Determined by fMRI, the dorsolateral prefrontal cortex (DLPFC)

is less active in obese patients which leads to overeating. In the case of a higher activity of the DLPFC, weight-loss and maintaining healthy weight in obese patients are more likely (Ester and Kullmann, 2022). The orbitofrontal cortex (OFC) is involved in the assessment of nutritive qualities of the food and it is suggested that decreased function of the OFC in patients with obesity could be responsible for addictive eating behavior (Saruco and Pleger, 2021). Furthermore, if the anterior cingulate cortex (ACC) is more active during a food-related inhibition task, the participants are leaner (Saruco and Pleger, 2021). Therefore, in this study we examined guanylate cyclase C (GC-C) expression in: BA9 (DLPFC), 11 (orbitofrontal cortex), 10 (anterior prefrontal cortex), and 32 (dorsal anterior cingulate area).

In the brain, the agonists of particulate (cell membrane) guanylate cyclase (GC-A, -B or -C) play a role in neuronal development, synaptic transmission and neuroprotection (Markerink-Van Ittersum et al., 1997). Plasma concentrations of brain natriuretic peptide (BNP), an agonist of GC-A, are in correlation to cognitive function in demented patients and are associated with increased risk of developing cognitive disorders (Naito et al., 2009). Activation of brain GC-C by systemic or locally applied (intracerebroventricular) guanylin peptides leads to satiety in laboratory animals (Valentino et al., 2011; Folgueira et al., 2016). The concentrations of pro-uroguanylin (pro-UGN) in plasma [precursor of GC-C agonist, uroguanylin (UGN)] are decreased in obesity (Rodríguez et al., 2016).

More recent research has shown a rapidly expanding family of hormones expressed and active in both the gastro-intestinal (GI) tract and the brain (Ferrini et al., 2009; Whissell et al., 2019). Apart from its function in the GI tract, ghrelin regulates satiety and energy homeostasis (Ferrini et al., 2009), cholecystokinin is involved in memory and cognition as well as in the development of anxiety disorders (Zwanzger et al., 2012; Whissell et al., 2019) and secretin, which regulates water and food homeostasis, motor learning, spatial memory, fear, and anxiety (Wang et al., 2019). Due to the blood-brain barrier, it is still not clear which of the effects could be attributed to the hormones delivered from the GI and which to locally produced hormones in the brain. A new member of this family is UGN, a 16-amino-acid polypeptide secreted after a meal from the enterochromaffin cells into the gut lumen, but also into the blood (Fan et al., 1996; Li et al., 1997). UGN exerts physiological function in intestine, kidney, and brain. Concertation of pro-UGN is decreased in plasma of obese people (Rodríguez et al., 2016) which might correspond to results obtained from obese mice which secret less UGN after a meal (Simões-Silva et al., 2013). However, the pro-UGN concentrations in blood did not differ in human subjects with obesity when compared to not obese subjects suggesting importance of possible difference in GC-C expression. Changing the food content does not change the postprandial increase in pro-UGN blood concertation (Patterson et al., 2020).

Even though UGN is an agonist of GC-C, its expression in the brain [cerebellum (Cb), hypothalamus (Hy), cerebral cortex, and midbrain (MB)] (Fan et al., 1997; Habek et al., 2023) is often contested and ignored (Kim et al., 2016).

GC-C is detected in the mouse cerebral cortex, amygdala (Amyg), MB, Hy, and cerebellar Purkinje cells (Gong et al., 2011; Valentino et al., 2011; Begg et al., 2014; Dugandzic et al., 2020; Habek et al., 2020, 2021). All these brain areas are, in one way or another, involved in feeding regulation (de Vrind et al., 2021; Low et al., 2021; Iosif et al., 2023). GC-C is expressed in dopaminergic neurons of the ventral tegmental area and substantia nigra (SN) where GC-C activation increases neuronal activity via potentiation of glutamate and acetylcholine effects suggesting that GC-C regulates dopamine release (Gong et al., 2011). Those GC-C positive neurons project to the nucleus caudatus and putamen, nucleus accumbens, olfactory tubercle, lateral hypothalamic area, and central nucleus of Amyg (Merlino et al., 2019).

GC-C is expressed in the neurons of the basolateral and cortical nucleus of Amyg, where it is involved in the regulation of anxiety-like behavior. In the Amyg, expression of GC-C mRNA increases 2 h after feeding in female mice, but not in male mice (Dugandzic et al., 2020). In addition to neurons expressing GC-C, the Amyg gets projections from GC-C positive neurons located in the Hy and MB (Merlino et al., 2019).

As we know so far, the main site of UGN metabolic effects is the Hy. GC-C is located in pro-opiomelanocortin (POMC) expressing neurons of the arcuate nucleus (Arc) of mice and humans (Habek et al., 2020). In addition, it is expressed in the ventral premammillary nucleus (PMV). The GC-C-positive neurons from the PMV project to the nucleus posterior of Amyg, ventral part of the lateral septal nucleus, nucleus of the stria terminalis, medial preoptic nucleus, Arc, and ventromedial nucleus (Merlino et al., 2019). Two hours after a meal, there is a lower expression of GC-C in Arc of the Hy in female mice compared to males, while the expression of GC-C is the same at fasting conditions (Habek et al., 2020).

The expression of GC-C in the human brain is confirmed in the hypothalamic Arc and PFC (Colantuoni et al., 2011; Habek et al., 2020). Since the expression in the female DLPFC (BA46 and BA9) decreases by age (Colantuoni et al., 2011) and the function of GC-C is dependent on sex and feeding status, aims of the study are to determine possible age and sex differences of GC-C expression in regions involved in feeding regulation: Brodmann areas (BA) 9 (DLPFC), BA11 (orbitofrontal cortex), BA10 (anterior prefrontal cortex), and BA32 (dorsal anterior cingulate area) in 21 male and 13 female brain samples with a postmortem delay less of 24 h (Brodmann, 1909; Banovac et al., 2020). Special attention is paid to the time passed between the subject's last meal and their time of death. Since previous research suggested differences in the regulation of eating behavior in the left and right hemispheres (Lowe et al., 2019), we also determined possible difference in GC-C expression in areas of interest in the left and right hemispheres. GC-C expression was confirmed in the Arc of the Hy, and, for the first time, it was detected in the human SN and the cerebellar cortex, which are areas known to be involved in feeding regulation.

## Materials and methods

### Tissue sampling

Human brain tissue was collected from 35 deceased persons (22 men and 13 women). The average age was 53 ± 3 years for men and of 64 ± 6 years for women (*p* = 0.08, difference not statistically significant). Brain tissue samples (Hy, MB, Cb, and PFC) from both hemispheres were collected during standard autopsy after obtaining a signed informed consent form from the deceased's next of kin. All personal data were stored under a generated code and processed electronically. The principal investigator and team members respected all regulations and standards for protection of personal information and the identity of the participants was not revealed to third persons. Differences in GC-C expression due to sex, age, and feeding status were determined in brain samples collected from 34 deceased subjects (Supplementary Table 1). The obesity was estimated by physician coroner during regular autopsies and equally distributed in both sexes (6 of 13 women and 10 of 21 men). Less than 100 mL of the stomach content was classified as an empty stomach (person died long after eating a last meal), and 100 mL or more of content found during autopsy was classified as a full stomach. Postmortem delay for all collected samples was under 24 h. After collection, half of the tissue for further protein isolation was frozen and stored at −80°C until further use.

The brain sample used for immunostaining was obtained from 40 years old men with no medical history of neurological or psychiatric disorders and no neuropathological deviations in the brain on autopsy. Relevant medical history was obtained from both autopsy reports and medical records. The analyzed subject died without a preagonal state, and the postmortem delay (6.5 h) represents the actual interval of neuron death. The brain tissue is a part of the Zagreb Neuroembryological Collection. It was obtained with the approval of the Ethics Committee of University of Zagreb School of Medicine (380–59-10106–14-55/152; Banovac et al., 2022).

### Protein isolation

Frozen tissue samples were thawed on ice and washed in ice-cold phosphate-buffered saline (PBS) solution. The desired areas of the PFC were sampled [BA: 9 (*n* = 28), 10-orbital (O, *n* = 29), 10-medial (M, *n* = 32), 11 (*n* = 30) and 32 (*n* = 28)], as well as SN (*n* = 22), Arc of Hy (*n* = 30) and cerebellar cortex (*n* = 33). BA9 was sampled from the dorsal part of the superior frontal gyrus, BA10-O was sampled from the ventral (orbital) aspect of the frontal pole, BA10-M was sampled from the ventro-medial part of the superior frontal gyrus, BA11 was sampled from the rostral part of the straight gyrus (gyrus rectus) and BA32 was sampled from the paralimbic cortex situated between the paracingulate sulcus and the cingulate gyrus. The samples were not taken if it was not possible to sample the brain region of interest with a great degree of certainty. The number of samples for SN is the smallest because the sample was only taken when the structure was clearly visible which was not the case in some elderly subjects. Approximately 100 mg of tissue was excised per region, placed in a labeled microcentrifuge tube (Thermo Fisher Scientific, Waltham, MA, USA) and ice-cold PBS was added (1 g tissue = 9 mL PBS). The tissue was thoroughly homogenized with an ultrasonic processor (Q55 Sonicator^®^, QSonica Sonicators, Newton, CT, USA). Samples were centrifuged (5 min, 5,000 *g*, +4°C; Eppendorf Centrifuge 5415 R, Hamburg, Germany). The supernatant was transferred into a new microcentrifuge tube (Thermo Fisher Scientific) and stored at −80°C until use.

### ELISA

GC-C expression was determined with a commercially available Enzyme-Linked Immunosorbent Assay kit (E5383Hu ELISA; Bioassay Technology Laboratory, Birmingham, UK) following the manufacturer's instructions. Briefly, all reagents and samples were brought to room temperature before use and standard solutions were prepared shortly before commencement. Standard solutions and samples (40 μl) were loaded into the provided 96-well plate pre-coated with the Human anti-GC-C antibody. Sample wells were then loaded with the biotinylated anti-GC-C antibody after which streptavidin-HRP (horseradish peroxidase) was added to all sample wells. The plate was incubated for 60 min at 37°C (Heratherm™ Compact Microbiological Incubator, Thermo Fisher Scientific). Excess streptavidin-HRP was then washed away (5 × 1 min) and substrate solutions were added to each well. Following a brief incubation (10 min, 37°C, dark environment) a stop solution was added and optical density was read using a microplate reader (GloMax^®^ Explorer Multimode Microplate Reader, Promega Corporation, Madison, WI, USA) set at 450 nm. A standard curve was generated from the results and used to determine GC-C expression in each sample.

### Tissue fixation

The brain tissue was cut into blocks following Talairach's coordinates (Talairach and Szikla, 1980). Tissue blocks of the DLPFC containing the superior frontal gyrus (BA9) were selected (Brodmann, 1909; Banovac et al., 2020). The tissue was first fixed by immersion in 4% paraformaldehyde (PFA; Biognost, Zagreb, Croatia) for 24 h, then dehydrated in an ethanol cascade (70, 96, and 100%; Biognost). Where needed, surface vessels were removed with tweezers to avoid tissue rupture during sectioning. Tissue was then incubated in toluene (2 × 4 h; Kemika d.d., Zagreb, Croatia) and lastly in paraffin (2 × 24 h; Biognost) before being embedded in paraffin blocks and stored at room temperature until use (Sadeghipour and Babaheidarian, 2019). Brain sample was cut on a microtome into 20-μm-thick coronal slices (Sy and Ang, 2019) and mounted on VitroGnost Plus Ultra adhesive microscope slides (BioGnost, Zagreb, Croatia).

### Immunohistochemistry

This part of the study was performed according to protocols for paraffin-embedded tissue (Zaqout et al., 2020). Histological sections were first photobleached for 48 h using a LED light source (Neumann and Gabel, 2002; Sun et al., 2017) in order to reduce autofluorescence. The sections were deparaffinized and heat antigen retrieval was performed in citrate-based (pH 6.0) unmasking solution (Boenisch, 2005) followed by protein blocking [1 h at room temperature (RT) in normal donkey serum (NDS; Chemicon, USA) diluted 1:30 in permeabilization solution (0.3% Triton X-100 in 1x PBS; Sigma-Aldrich, USA)]. The sections were then incubated overnight in primary antibodies at 4°C. After washing (3 times per 10 min in PBS), the sections were incubated with secondary antibodies for 1 h at room temperature. Primary and secondary antibodies used for immunofluorescence are shown in Table 1. To further reduce autofluorescence, brain sample was treated with TrueBlack^®^ Lipofuscin Autofluorescence Quencher (Biotium, USA; Banovac et al., 2019, 2022) and coverslipped with VECTASHIELD^®^ Antifade Mounting Medium (Vector Laboratories, USA). Histological sections were imaged using a laser confocal microscope (Olympus FLUOVIEW FV3000RS, Japan) on high-power magnification and using *Z*-stack in order to visualize the entire section thickness.

**Table 1:** Primary and secondary antibodies used for immunofluorescence.

**Antibody**	**Species and clonality**	**Manufacturer, catalog number (CN), lot**	**Working dilution**
**Primary antibodies**			
Anti-NeuN	Rabbit; polyclonal	Abcam: CN: ab104225; lot: GR3370892-1	1:1000
Anti-GC-C (GUCY2C)	Mouse; monoclonal	US Biological Life Sciences: CN: 207689; lot: L21102855	1: 200
**Secondary antibodies**			
Conjugated anti-mouse Alexa 488	Donkey	ThermoFisher (Invitrogen): CN: A-21202; lot: 1915874	1:1000
Conjugated anti-rabbit Alexa 546	Donkey	ThermoFisher (Invitrogen): CN: A10040; lot: 1833519	1:1000

### Statistical analyses

To test the normal distribution of the results we used the Kolmogorov-Smirnov test. When expression of GC-C is compared we used unpaired Student's *t*-tests. To determine possible correlations between age and GC-C expression or between different brain regions we used Pearson's correlation test. The data was presented as mean ± standard error mean (SEM) and *p* < 0.05 was considered statistically significant. For statistical analyses the GraphPad Instat statistical software (Graph-Pad Software, Boston, MA, USA) was used.

## Results

In this study we determined the expression of GC-C in male and female Brodmann areas (BA) 9, 10 (O—orbital and M—medial), 11, 32, the Arc of the Hy, the SN and the cerebellar cortex. In his study we determine the expression of GC-C in correlation to feeding status determined as a volume of stomach content during autopsy, sex differences in GC-C expression and differences in GC-C expression in left compared to right hemisphere.

### Expression of GC-C depends on feeding status

**Figure 1:**
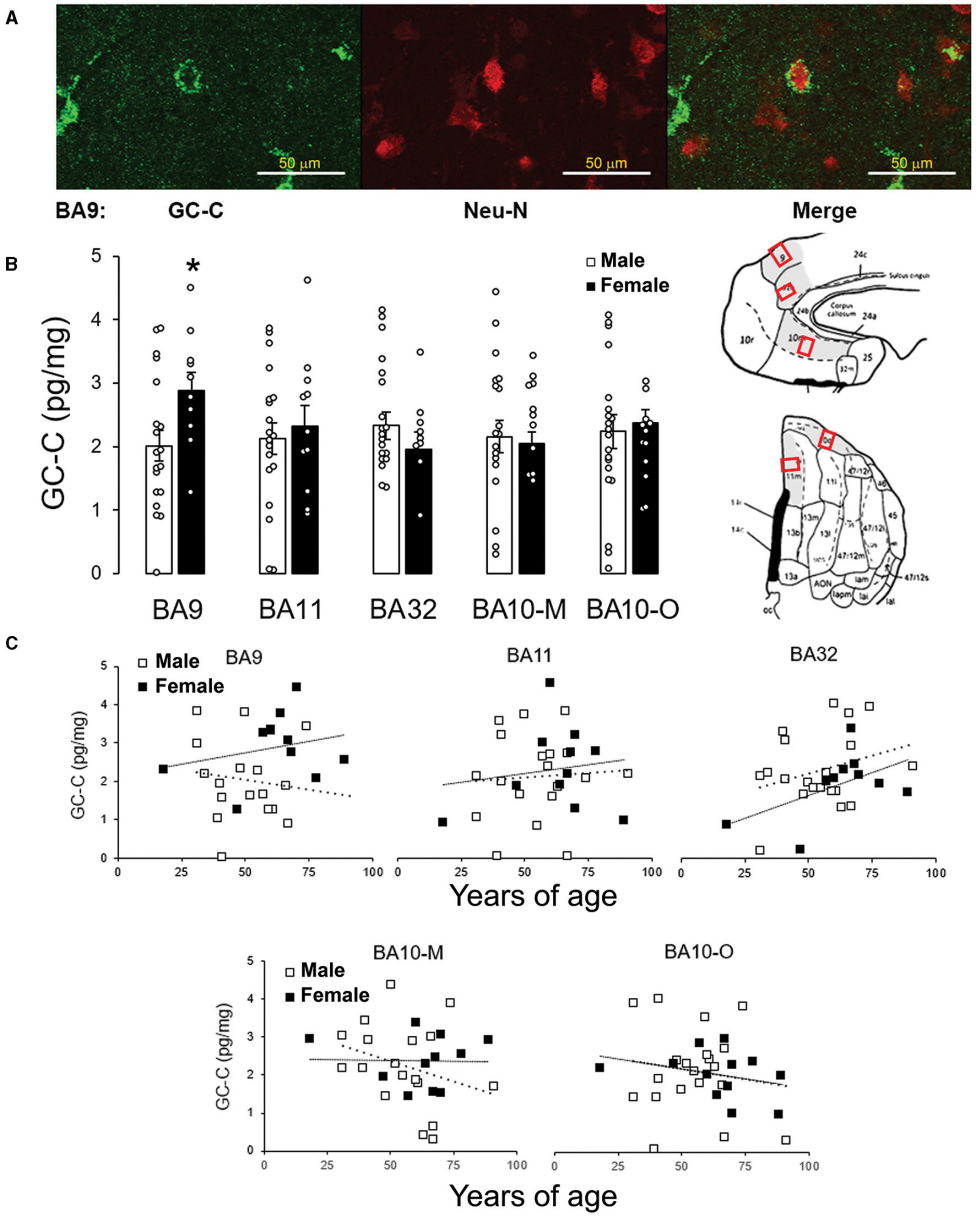
GC-C is more expressed in female than male BA9. GC-C is present in neurons in layer III of BA9. GC-C is presented as green and Neu-N (neuronal marker) as red. Bar represents 50 μm **(A)**. When the expression of GC-C was compared, of all tested areas the difference between male and female brains is found in BA9. Results are presented as mean ± SEM (pg/mg of tissue; *p < 0.05 statistically significant) **(B)**. Regions of interest are gray on the BA Maps (Ongür and Price, 2000; Ongür et al., 2003) and exact sampling location was presented by red squares. The expression of GC-C is not age dependent in men's and women's cortical regions. Results are presented as pg/mg of tissue. - - - line represents trendline for men. …… line represents trendline for women **(C)**. BA, Brodmann area; GC-C, guanylate cyclase C.

The expression of GC-C in BA9 was confirmed by immunohistochemical staining. GC-C was located in neurons of the layer III of man's BA9 prefrontal cortical area. The neuronal localization was confirmed by co-localizing with the neuronal marker Neu-N (Figure 1A). Statistically significant correlation between stomach volume determined during autopsy and expression of GC-C was found in BA11 and BA10-M. A volume of stomach content was negatively associated with GC-C expression in male BA11 and BA10-M (*r* = −0.61, *p* = 0.015 and *r* = −0.52, *p* = 0.038, respectively), while there was no significant correlation in female BA11 (*r* = −0.03, *p* = 0.941). Interestingly, in female 10-M there was a significant positive correlation between stomach content volume and GC-C expression (*r* = 0.80, *p* = 0.010) (Table 2).

**Table 2:** Correlation between GC-C expression and volume of stomach content.

**GC-C/stomach content**	**Hy**	**MB**	**Cb**	**BA9**	**BA11**	**BA32**	**BA10-M**	**BA10-O**
**Male**								
*r*	**−0.503**	−0.209	−0.039	−0.374	**−0.614**	0.200	**−0.523**	0.207
*p*	**0.040**	0.493	0.878	0.187	**0.015**	0.442	**0.038**	0.425
**Female**								
*r*	−0.555	−0.402	−0.011	0.316	0.029	0.001	**0.796**	−0.299
*p*	0.153	0.423	0.976	0.407	0.941	0.998	**0.010**	0.434

BA, Brodmann area; Cb, cerebellar cortex; Hy, hypothalamic Arcuate nucleus; O, orbital; M, medial. Bold values indicate statistically significant correlation when *p* < 0.05.

### Expression of GC-C is higher in female than male BA9

The sex difference was found in BA9 (Figure 1B). Even though, previous study showed negative correlation between expression of mRNA for GC-C in dorsolateral prefrontal cortex (BA46/9) with age in the female brain (Colantuoni et al., 2011), at the protein level there was no statistically significant correlation between age and GC-C expression for all tested cortical regions (Figure 1C).

**Figure 2:**
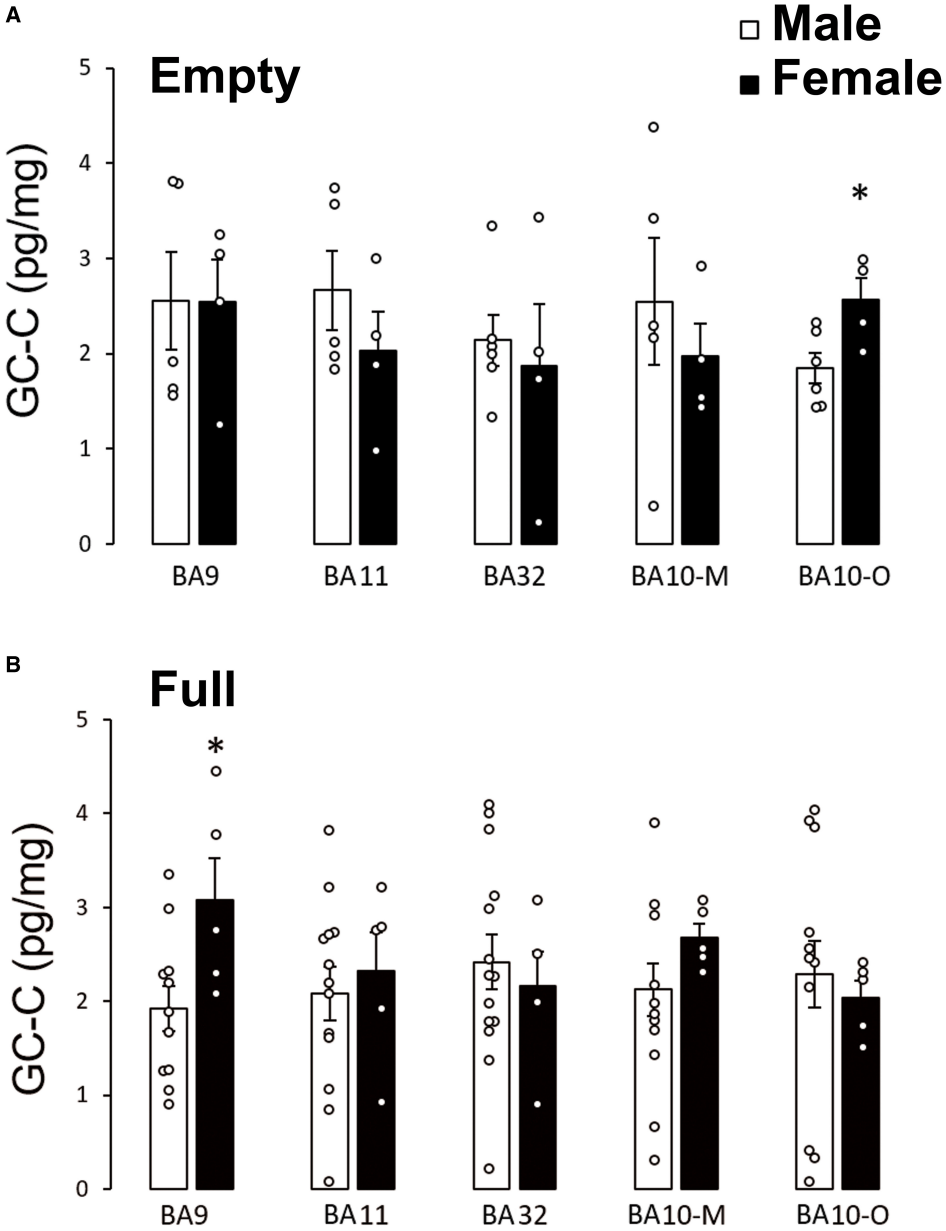
Sex differences of GC-C expression in the prefrontal cortex depended on feeding status. Sex differences in GC-C expression existed in BA10-O only if the stomach was empty (fasting conditions) in which case more GC-C is expressed in female participants **(A)**. Sex difference in BA9 exists if the stomach was full **(B)**. *p < 0.05 statistically significant. The results are presented as mean ± SEM (pg/mg of tissue). BA, Brodmann area; GC-C, guanylate cyclase C.

To determine if feeding status can affect sex differences of GC-C expression in human prefrontal cortex, we compared GC-C expression in cortical regions when the stomach was empty or full during autopsy. Surprisingly, in fasting conditions there was no difference in GC-C expression between male and female BA9 (Figure 2A). Sex difference in GC-C expression in BA10-O existed only if the stomach was empty (Figure 2B).

### Expression of GC-C in right and left hemisphere differs only in female brains

**Figure 3:**
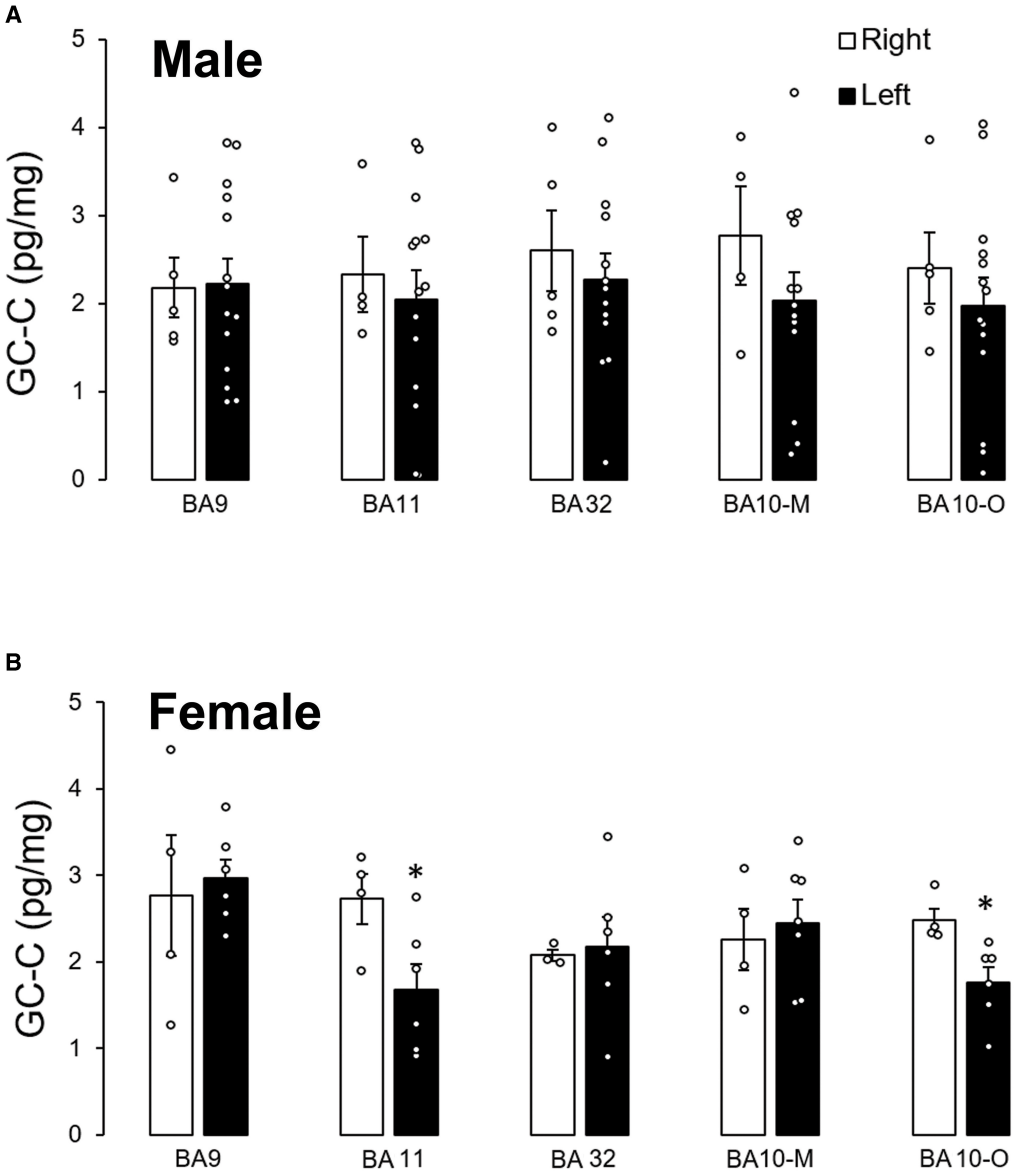
Expression of GC-C in right and left hemisphere was different only in female participants. There is no difference in GC-C expression between left and right male cortical regions **(A)**. Expression of GC-C in female BA11 and BA10-O is smaller in left hemisphere (*p < 0.05) **(B)**. The results are presented as mean ± SEM (pg/mg of tissue). BA, Brodmann area; GC-C, guanylate cyclase C.

There was no statistically significant difference in GC-C expression between male left and right hemispheres in any of the examined cortical regions (Figure 3A). However, when the stomach was empty there was more than a two times higher expression of GC-C in the left than the right BA9 (right: 172 ± 11, *n* = 3; left: 381 ± 1 ng/L, *n* = 2; *p* = 0.0007). After a meal, the expression in the left BA9 decreased (192 ± 32 ng/L, *n* = 8; *p* = 0.02) compared to the left BA9 before meal. Furthermore, differences in GC-C expression between the left and right hemisphere were determined in female BA11 and BA10-O where GC-C had a higher expression in the right than in the left hemisphere (Figure 3B).

### GC-C expression in arcuate nucleus of hypothalamus is lower in female than male

To determine further sex difference in the human brain, we determined GC-C expression in the Arc of the Hy, the SN and the cerebellar cortex. Statistically significant negative correlation between the volume of stomach content and GC-C expression in the Arc of the Hy was found in male subjects (*r* = −0.503, *p* = 0.040), while this correlation was not statistically significant in female subjects (*r* = −0.555, *p* = 0.153, Table 2).

**Figure 4:**
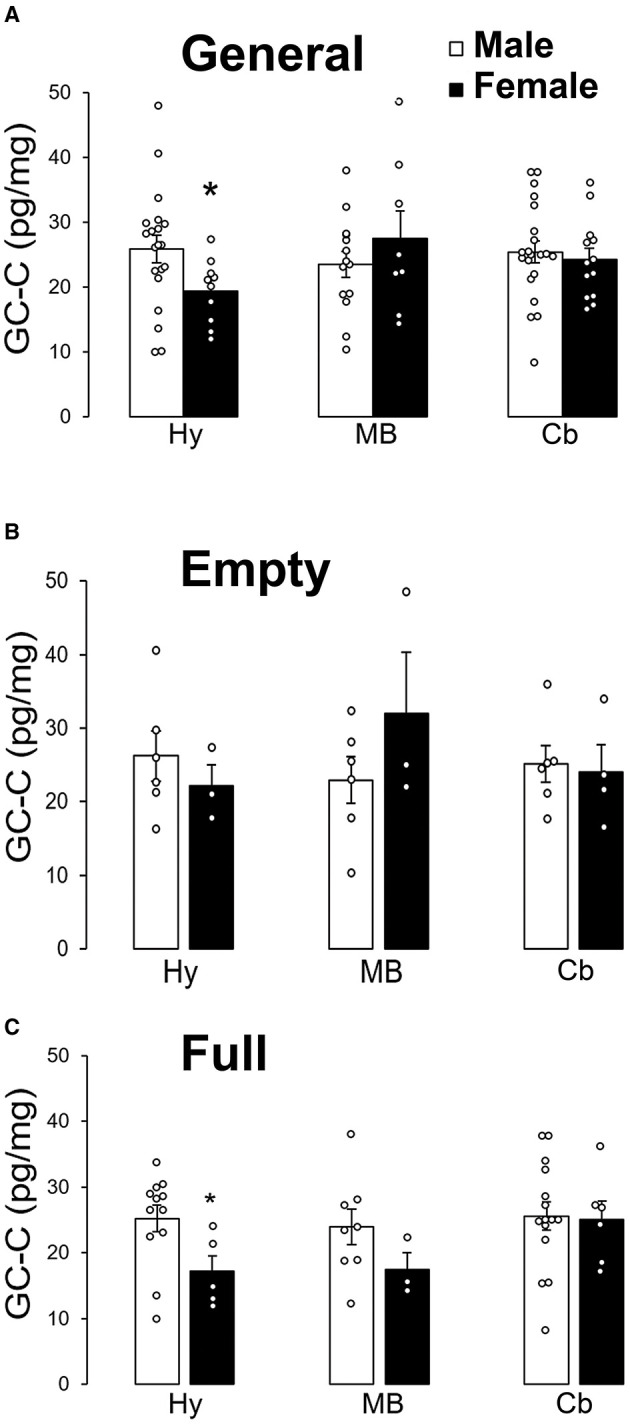
GC-C is less expressed in the female arcuate nucleus of the hypothalamus after a meal. GC-C is less expressed in female arcuate nucleus (Hy) than in male. Expression of GC-C in midbrain (MB, substantia nigra) and cerebellar cortex (Cb) did not statistically significantly differ between male and female participants **(A)**. There is no statistically significant difference in the GC-C expression in all tested regions of the brain when the stomach was empty **(B)**. Sex difference of GC-C expression in the hypothalamus only exists when the stomach was full **(C)**. *p < 0.05 statistically significant. The results are presented as mean ± SEM (pg/mg of tissue).

GC-C was less expressed in female Arc than in male. There was no statistically significant sex difference in GC-C expression in the SN (MB) or the cerebellar cortex (Figure 4A). Sex difference in GC-C expression was not present when the stomach was empty (Figure 4B), but was present when the stomach was full (Figure 4C).

**Figure 5:**
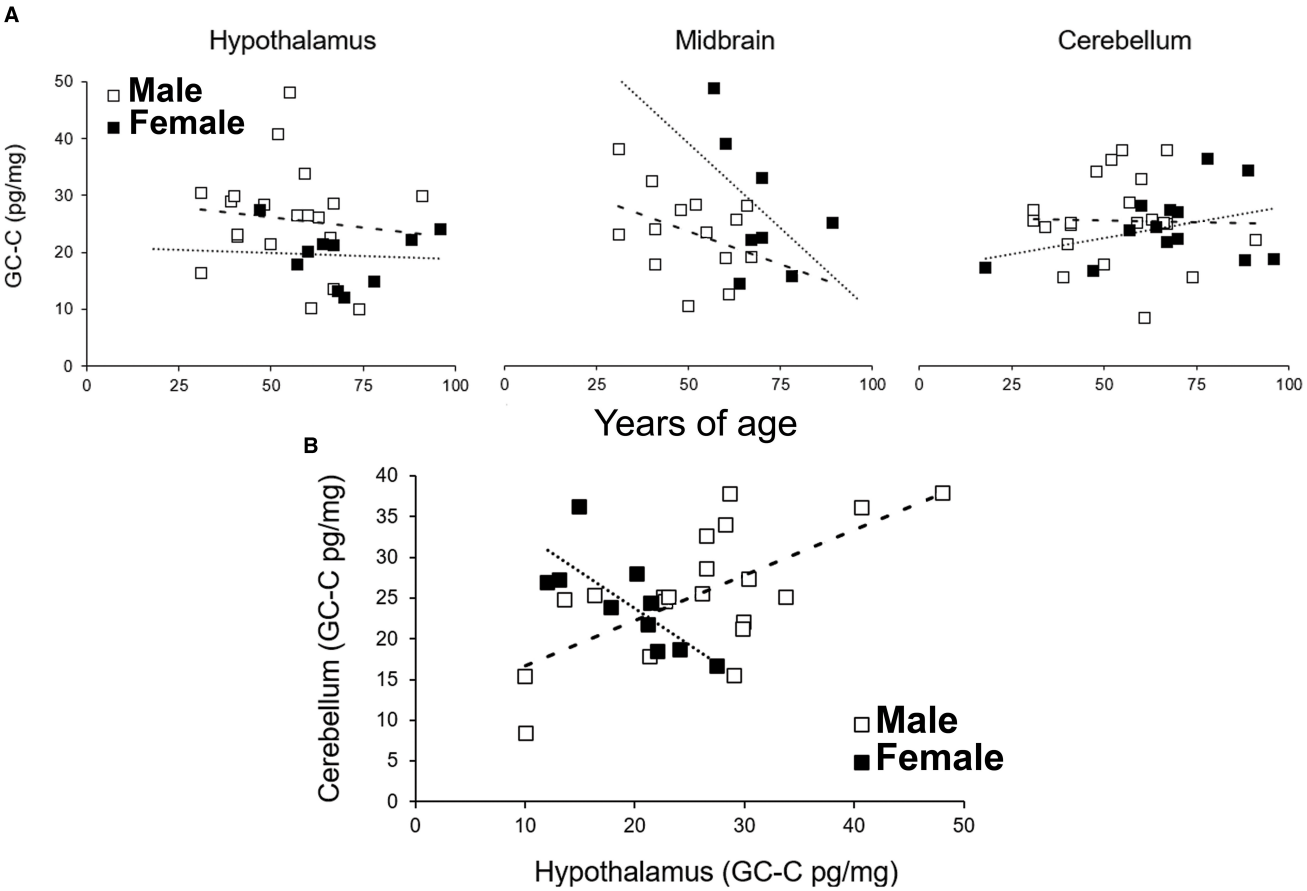
The expression of GC-C in the hypothalamus, midbrain and cerebellum is not correlated with age **(A)**. The expression of GC-C in the hypothalamus was in positive correlation to expression in cerebellar cortex in men, while it was in negative correlation in female participants **(B)**. Results are presented as pg/mg of tissue. - - - line represents trendline for male participants. …… line represents trendline for female participants. GC-C, guanylate cyclase C.

The GC-C expression did not depend on age. Even though there was a possible negative correlation between age and GC-C expression in the female MB, this correlation was not statistically significant (Figure 5, *r* = −0.51, *p* = 0.19). GC-C was statistically significantly expressed more in female MB when compared to male but only until the age of 60 (expression in male SN is only 55% of female SN, *p* = 0.009). In older age this difference was not observed.

Furthermore, the expression of GC-C in the Cb was in positive correlation to the expression in the male Hy (*r* = 0.67, *p* = 0.001) but in negative correlation in female Hy (*r* = −0.76, *p* = 0.01, Figure 5B), which also suggested a difference in the expression and regulation of GC-C in the male and female brain.

In this study we did not find statistically significant correlation in GC-C expression between BA9, BA10, BA11, and BA32 and Hy, MB, or Cb (Supplementary Figure 1). When the results were analyzed by including feeding status, there was a negative correlation between expression of GC-C in the Cb compared to the expression in male BA11 when the stomach was empty (*r* = −0.94, *p* = 0.016). The negative correlation was also determined for female BA9 and Hy when the stomach was full. On the other hand, positive correlation was determined in GC-C expression in male BA9 and the Cb when the stomach was full (Supplementary Table 2). Those results suggest the importance of feeding status and sex in regulation of GC-C expression in different brain regions.

## Discussion

Recent research points out the biological basis of sex differences in eating disorders (Culbert et al., 2021). Brian functions of estrogens on feeding regulation may be, at last partially, responsible for differences in food intake between men and women and higher occurrence of eating disorders in young women (Sodersten and Bergh, 2003). Additionally, to hunger and satiety regulation by the Hy, eating behavior is emotionally and motivationally regulated by the limbic system, Amyg and PFC. GC-C positive neurons are not found only in subcortical structures (MB, Amyg, Arc of Hy) but also, as we showed in this study, in the PFC. In humans such neurons are present especially in part of the cortex involved in regulation of feeding behavior. In this study we examined age, sex, and feeding status differences of GC-C expression in: BA9 (DLPFC), 11 (orbitofrontal cortex), 10 (anterior prefrontal cortex), 32 (dorsal anterior cingulate area), Arc of Hy, SN (MB), and cerebellar cortex. Since GC-C regulates feeding behavior in laboratory animals (Valentino et al., 2011; Folgueira et al., 2016), the differences in GC-C expression after a meal in comparison to fasting condition is not surprising. Sex differences in GC-C function is known in satiety and feeding regulation, brown adipose tissue activity and kidney function in laboratory animals (Dugandzic et al., 2020; Habek et al., 2020), therefore it is not surprising that the sex difference in GC-C expression exists as well in the human PFC.

Colantuoni et al. (2011) showed decreasing the expression of mRNA for GC-C with age in female DLPFC (BA46/9), but at the protein level there was no statistically significant correlation between age and GC-C expression for all tested cortical regions. In addition to differences in levels of expression of mRNA or protein, possible difference might be in the age span of our participants (18–96 years of age), since this study included only adults.

The regulation of GC-C expression by feeding status was found in male BA11 and BA10-M where GC-C expression was in negative correlation to volume of stomach content during autopsy. In female BA11 there was no detected correlation. On the other hand, in BA10-M the correlation was positive, which indicates sex differences in the regulation of GC-C, which in turn points to the regulation of BA11 and BA10-M function by feeding. The lack of GC-C regulation by feeding in female BA11 may contribute to the higher prevalence of eating disorders (Suda et al., 2010). To the best of our knowledge, this is the first study which compares the expression of proteins in the brain in relation to the feeding status during death by measuring the stomach content at autopsies. This can be a useful tool of determining the importance of proteins of interest in feeding regulation especially in the human PFC.

The expression of GC-C is regulated differently in male and female BA9. When the stomach is full, there is a higher expression of GC-C in women compared to men. These results are in accordance with a previously published study which shows that after showing images of hedonic foods to male and female participants, there is a greater activation of DLPFC in women when compared to men. Difference in activity was not shown when participants were hungry (Cornier et al., 2010). Higher expression of GC-C in female BA9 after a meal might be involved in greater activation of PFC which regulates executive functioning and inhibitory control after consumption of palatable food (Del Parigi et al., 2002; Cornier et al., 2010; Geliebter et al., 2013).

When the stomach is empty there is higher expression of GC-C in left then right male BA9 which decreased after a meal. This regulation was not observed in female BA9. Changing the eating behavior toward healthy food choices increased left DLPFC activity. The capability of left DLPFC activation seems to be a predictor for successful weight loss in women (Le et al., 2006; Ester and Kullmann, 2022). The regulation of GC-C expression in the left DLPFC could be involved in left DLPFC activation as response to a meal. The right PFC is also responsible for the regulation of feeding behavior (Ester and Kullmann, 2022), however, further research is needed to determine the possible regulation of GC-C expression in right hemisphere.

If the orbitofrontal cortex is more active when eating fats that person will consume more fats (Khorisantono et al., 2023) which might contribute to the obesity development. This brain region is a part of the brain reward system and it receives dopaminergic afferents from the MB where it is affected by GC-C (Schultz et al., 2000; Saper et al., 2002; Knutson et al., 2003; Heekeren et al., 2004; Cox et al., 2005; Gong et al., 2011). Eating behavior in persons with eating disorders is connected to the function of the left orbitofrontal cortex (Suda et al., 2010). Difference in GC-C expression between the left and right hemispheres was determined in female BA11, whereas GC-C is more expressed in the right than in the left hemisphere.

BA10 is involved in food evaluation process (Hollmann et al., 2013). Using fMRI, after looking a food pictures activities of left male BA10 decreased (Yoshikawa et al., 2016). In female left BA10 chronic stress reduced activity upon looking the picture of high calorie foods which might lead to obesity (Tryon et al., 2013). To determine the possible differences in regulation of GC-C expression in BA10, we separated this region into BA10-O and BA10-M. As shown for BA9, when the stomach is full, the higher expression of GC-C is found in female BA10-O, but only when the stomach is empty. Differences in GC-C expression between left and right hemisphere was determined in female BA10-O whereas GC-C is more expressed in men's the right than the left hemisphere, while there is no difference in men's either part of BA10.

BA32 (dorsal anterior cingulate area) is an integral part of the limbic system and is involved in the regulation of eating behavior (Zhang et al., 2023). In this study we found no statistically significant difference between male vs. female, left vs. right or feeding status regulation of GC-C expression in BA32.

The sex differences and feeding status are important for the regulation of GC-C expression in subcortical structures as well. As shown for the mouse Hy (Habek et al., 2020), sex difference in GC-C expression exist when the stomach is full, suggesting possibly lower satiety effects of GC-C agonists in women. The negative correlation exists in GC-C expression in female but not male BA9 and Hy when the stomach is full suggesting a different regulation of GC-C by feeding in the Hy and PFC with possibility that this regulation is sex dependent.

After activation either by glucose via glucose-sensitive neurons or via vagus from the intestine, VTA releases dopamine—the pleasure hormone. Dopamine release from dopaminergic neurons in the MB is potentiated by the activation of GC-C (Gong et al., 2011). Mice missing GC-C (GC-C KO) develop attention deficit hyperactivity disorder (ADHD; Gong et al., 2011). GC-C is expressed more in female MB in relation to male but only until the age of 60. These findings could potentially contribute in clarification of the higher prevalence of ADHD in younger men. Dopamine in the nucleus accumbens leads to the creation of feelings of happiness and satisfaction. The nucleus accumbens sends information to the Amyg that attributes to the experienced reward. Furthermore, by prediction of feeding, search for a food, and eating more dopamine is released in prefrontal cortex (Horvitz, 2000; Phillips et al., 2004). It is not surprising that GC-C is expressed in MB, PFC, and Amyg and its expression is higher in those regions of female brain (Dugandzic et al., 2020) which might contribute to their activation (executive functioning, inhibitory control and reward) after a meal.

To conclude, the regulation of PFC function by feeding includes sex dependent regulation of GC-C expression (BA9, BA11, BA10-O, and BA10-M). GC-C expression differs in the left and right hemispheres but only in female BA11 and BA10-O. For tested subcortical structures, the expression of GC-C in the female Hy was lower in relation to male, but only when the stomach was full. These results suggest possible role of GC-C in the regulation of feeding behavior and existing sex differences in this regulation. Further research is needed to reveal the connection between GC-C positive neurons in subcortical structures (Hy, Amyg, and SN) and PFC, and the importance of GC-C regulation of dopamine release, as well as its possible importance in eating disorders. Comparing the expression of proteins in relation to feeding status by measuring the stomach content at autopsies will give us better understanding how the PFC is involved in regulation of feeding behavior. Since the majority of the studies have been performed at men or even in mixed population, we have to pay more attention to sex differences for determining the specific role of PFC in regulation of eating and eating disorders.

## Data availability statement

The raw data supporting the conclusions of this article will be made available by the authors, without undue reservation.

## Ethics statement

The studies involving humans were approved by Ethical Committee of the University of Zagreb School of Medicine (641-01/18-02/01). The studies were conducted in accordance with the local legislation and institutional requirements. The human samples used in this study were acquired during standard autopsy after obtaining a signed informed consent form from the deceased's next of kin.

## Author contributions

MR: Formal analysis, Methodology, Validation, Writing—review & editing, Data curation. VC: Data curation, Formal analysis, Methodology, Writing—review & editing. MT: Data curation, Formal analysis, Methodology, Writing—review & editing. AM: Data curation, Formal analysis, Methodology, Writing—review & editing. PB: Conceptualization, Data curation, Formal analysis, Methodology, Writing—review & editing. PŠ: Conceptualization, Data curation, Formal analysis, Methodology, Supervision, Writing—review & editing. IB: Conceptualization, Data curation, Formal analysis, Investigation, Methodology, Supervision, Writing—review & editing. AD: Conceptualization, Formal analysis, Funding acquisition, Investigation, Methodology, Project administration, Resources, Supervision, Validation, Visualization, Writing—original draft, Writing—review & editing.
