# Supplementary material for: Expression of guanylate cyclase C in human prefrontal cortex depends on sex and feeding status
Source: Front Mol Neurosci. 2024 May 22;17:1361089. doi: 10.3389/fnmol.2024.1361089 (PMC11150535; doi:10.3389/fnmol.2024.1361089)

Table S1: Brian samples were taken from following deceased participants.

No.	M/W	Body weight	Age (years)	Cause of death
1	Female	Normal	88	Heart failure
2	Female	Normal	96	Heart failure
3	Female	Obese	64	Heart failure
4	Male	Obese	57	Heart failure
5	Female	Obese	70	Drowning
6	Male	Obese	59	Heart failure
7	Male	Obese	34	Electric shock
8	Male	Normal	39	Acute poisoning
9	Male	Normal	67	Thromboembolism of pulmonary arteries
10	Male	Normal	55	Heart failure
11	Male	Obese	48	Choking on a bite of food
12	Male	Obese	52	Heart failure
13	Male	Obese	63	Heart failure
14	Female	Normal	18	Heart failure
15	Male	Normal	61	Acute poisoning
16	Female	Obese	47	Thromboembolism of pulmonary arteries
17	Male	Normal	91	Suicide
18	Male	Normal	41	Suicide
19	Male	Obese	41	Heart failure
20	Female	Normal	60	Brain haemorrhage
21	Male	Normal	50	Suicide
22	Male	Normal	31	Hear failure and pneumonia
23	Male	Obese	74	Choking on a bite of food
24	Male	Normal	40	Acute poisoning
25	Male	Normal	67	Internal bleeding
26	Male	Normal	66	Heart failure
27	Female	Normal	57	Heart failure
28	Female	Obese	67	Heart failure
29	Female	Obese	70	Fracture of the 2nd cervical vertebra
30	Female	Normal	89	Severe atherosclerosis, multiple injuries
31	Male	Obese	60	Cardiac tamponade
32	Female	Normal	78	Massive haemorrhages
33	Male	Obese	31	Suicide
34	Female	Obese	68	Heart failure

Table S2: Correlation in GC -C expression between prefrontal cortex and Hypothalamus (Hy), substantia nigra (MB) or cerebellum (Cb) is gender and feeding status dependent

Male (empty)	BA	9	11	32	10-M	10-O
Hy	r=	-0.703	0.299	0.039	-0.098	0.626
	p=	0.186	0.625	0.941	0.876	0.184
MB	r=	-0.528	-0.121	0.379	-0.435	0.132
	p=	0.360	0.847	0.459	0.464	0.803
Cb	r=	-0.499	**-0.943**	-0.343	-0.504	0.702
	p=	0.392	**0.016**	0.506	0.387	0.120

Male (full)	BA	9	11	32	10-M	10-O
Hy	r=	0.384	0.464	-0.282	0.345	-0.140
	p=	0.308	0.151	0.401	0.298	0.665
MB	r=	0.492	0.066	-0.455	0.368	0.563
	p=	0.262	0.888	0.257	0.370	0.146
Cb	r=	**0.609**	0.013	-0.199	-0.343	-0.078
	p=	**0.047**	0.967	0.495	0.251	0.792

Female (empty)	BA	9	11	32	10-M	10-O
Hy	r=	-0.967	-0.911	-0.692	0.989	-0.870
	p=	0.163	0.272	0.513	0.096	0.328
MB	r=	0.649	0.738	-0.453	-0.466	0.306
	p=	0.551	0.472	0.701	0.691	0.802
Cb	r=	0.415	-0.548	0.248	0.733	-0.471
	p=	0.585	0.452	0.752	0.267	0.529

Female (full)	BA	9	11	32	10-M	10-O
Hy	r=	0.063	**-0.966**	-0.461	0.951	-0.648
	p=	0.937	**0.034**	0.539	0.200	0.342
MB	r=	0.607	0.838	0.888	0.983	0.547
	p=	0.585	0.368	0.304	0.118	0.631
Cb	r=	-0.133	0.770	0.465	-0.319	0.271
	p=	0.832	0.128	0.430	0.601	0.660

BA: Brodmann area

Figure S1: No statistically significant correlation in GC-C expression between Brodmann areas (BA) 9, 10, 11 and 32 and hypothalamus, midbrain or cerebellum. BAs. - - - line represents trendline for male participants. ...... line represents trendline for female participants. GC-C - guanylate cyclase C

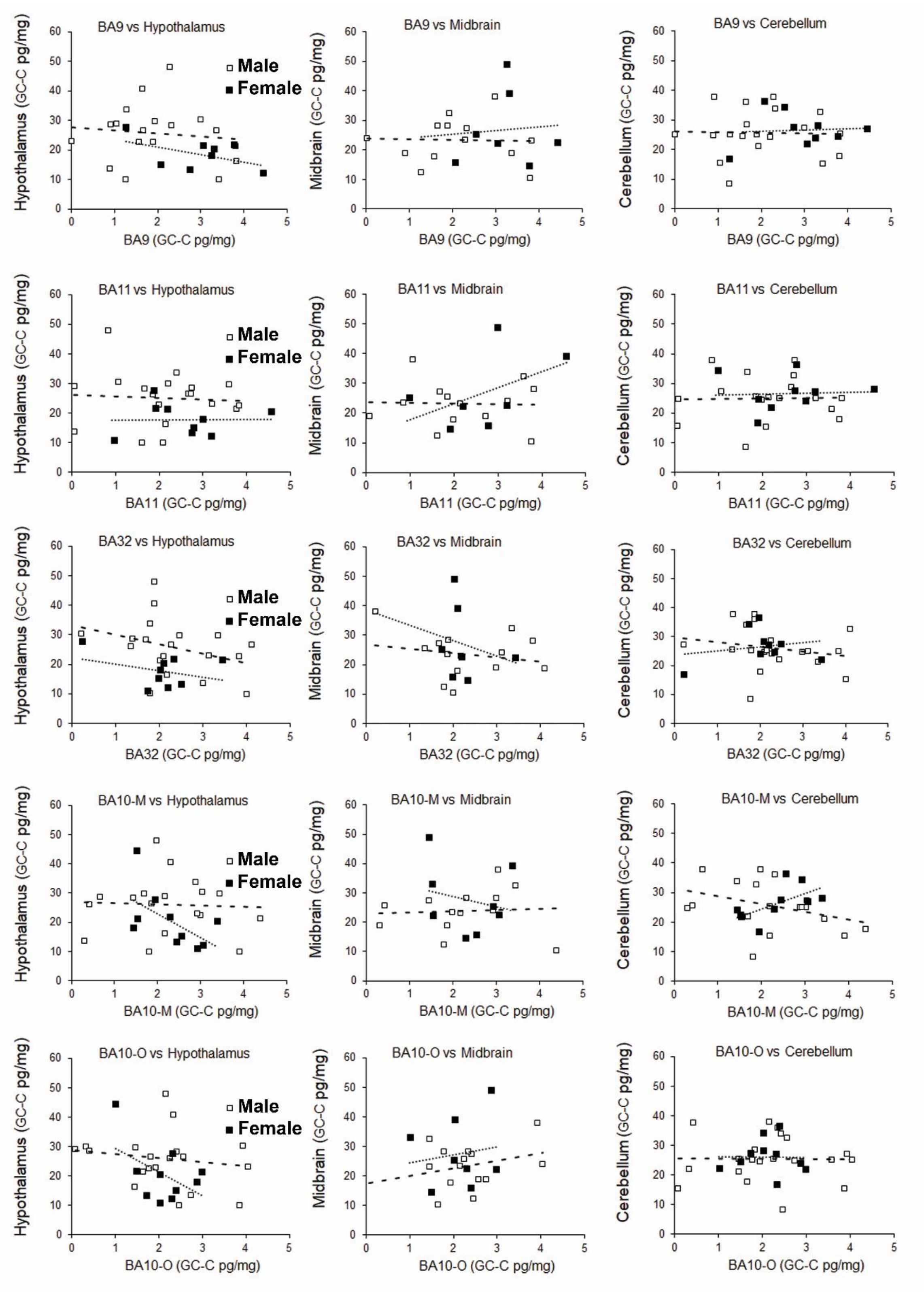

Supplement: Supplementary file 1 [file Data_Sheet_1.PDF]
